# Does adding a high-resolution 3D sequence improve the accuracy of erosion detection in pediatric sacroiliac joint MRI?

**DOI:** 10.1007/s00330-026-12709-y

**Published:** 2026-06-27

**Authors:** Nele Herregods, Robert G. W. Lambert, Stephanie Wichuk, Eva Schiettecatte, Thomas Renson, Joke Dehoorne, Lennart B. O. Jans, Jacob L. Jaremko

**Affiliations:** 1 Department of Radiology and Nuclear Medicine, Ghent University Hospital, Ghent, Belgium; 2 Department of Radiology & Diagnostic Imaging, University of Alberta, Edmonton, Alberta Canada; 3 Medical Imaging Consultants, Edmonton, Alberta Canada; 4 Department of Pediatric Rheumatology, Ghent University Hospital, Ghent, Belgium

**Keywords:** Juvenile idiopathic arthritis, Imaging, three-dimensional—sacroiliac joints, Sacroiliitis, Spondyloarthropathies

## Abstract

**Objective:**

To evaluate whether adding an erosion-sensitive high-resolution 3D sequence to sacroiliac joint (SIJ) MRI protocols in children improves erosion detection and diagnosis of Juvenile Spondyloarthritis (JSpA).

**Materials and methods:**

SIJ-MRI were prospectively obtained in 84 children (50 girls, mean age 13.3 y) clinically suspected of JSpA, conforming to 2024 ASAS/SPARTAN recommendations including a volumetric interpolated breath-hold examination (VIBE) sequence. Three experienced radiologists recorded the presence of erosions, non-erosion bony defects, and the presence of JSpA, with confidence scoring for each, in 3 blinded re-randomized reading exercises: (1) T1-weighted spin-echo (T1) and short-tau inversion recovery (STIR) sequences only; (2) VIBE only; (3) all sequences. Discrepancies were resolved by consensus read-out. The reference standard was rheumatologist diagnosis of JSpA.

**Results:**

MRI was considered indicative of JSpA in 14 cases (16.7%) on T1/STIR, 21 (25.0%) on VIBE only, and 15 (17.9%) with all sequences. In the three exercises, erosions were scored in 12 (14.3%), 22 (26.2%), and 13 (15.5%) cases, and non-erosion bony defects in 43 (51.2%), 79 (94.0%), and 80 (95.2%) cases. Sensitivity/specificity for JSpA diagnosis was 34.6/91.4%, 42.3/82.8%, and 38.5/91.4%, after consensus read-out 38.5/94.8%. Mean absolute values of confidence scores significantly improved for all features after adding VIBE, although inter-reader agreement did not consistently improve.

**Conclusion:**

Adding VIBE to T1/STIR significantly increased reader confidence in JSpA diagnosis. However, a trend toward improved sensitivity and specificity was not statistically significant. If the ASAS-SPARTAN SIJ MRI protocol is applied to children, all sequences must be interpreted together to avoid mistaking non-erosion defects for true erosions.

**Key Points:**

***Question*** *Can the recently published ASAS-SPARTAN adult standardized image acquisition protocol for sacroiliac joint MRI be used for diagnostic evaluation of sacroiliitis in children suspected for Juvenile Spondyloarthropathy (JSpA)?*

***Findings*** *The ASAS/SPARTAN recommendations including a high-resolution 3D erosion-sensitive sequence can also be applied to children, resulting in significantly increased reader confidence, with a trend toward improved sensitivity/specificity for diagnosing JSpA, although not statistically significant.*

***Clinical relevance*** *The ASAS-SPARTAN adult sacroiliac joint MRI protocol can also be beneficially applied to children, as long as all sequences are interpreted together to avoid mistaking non-erosion defects (almost universally seen on the 3D erosion-sensitive sequences in children) for true erosions.*

**Graphical Abstract:**

**Figure:**
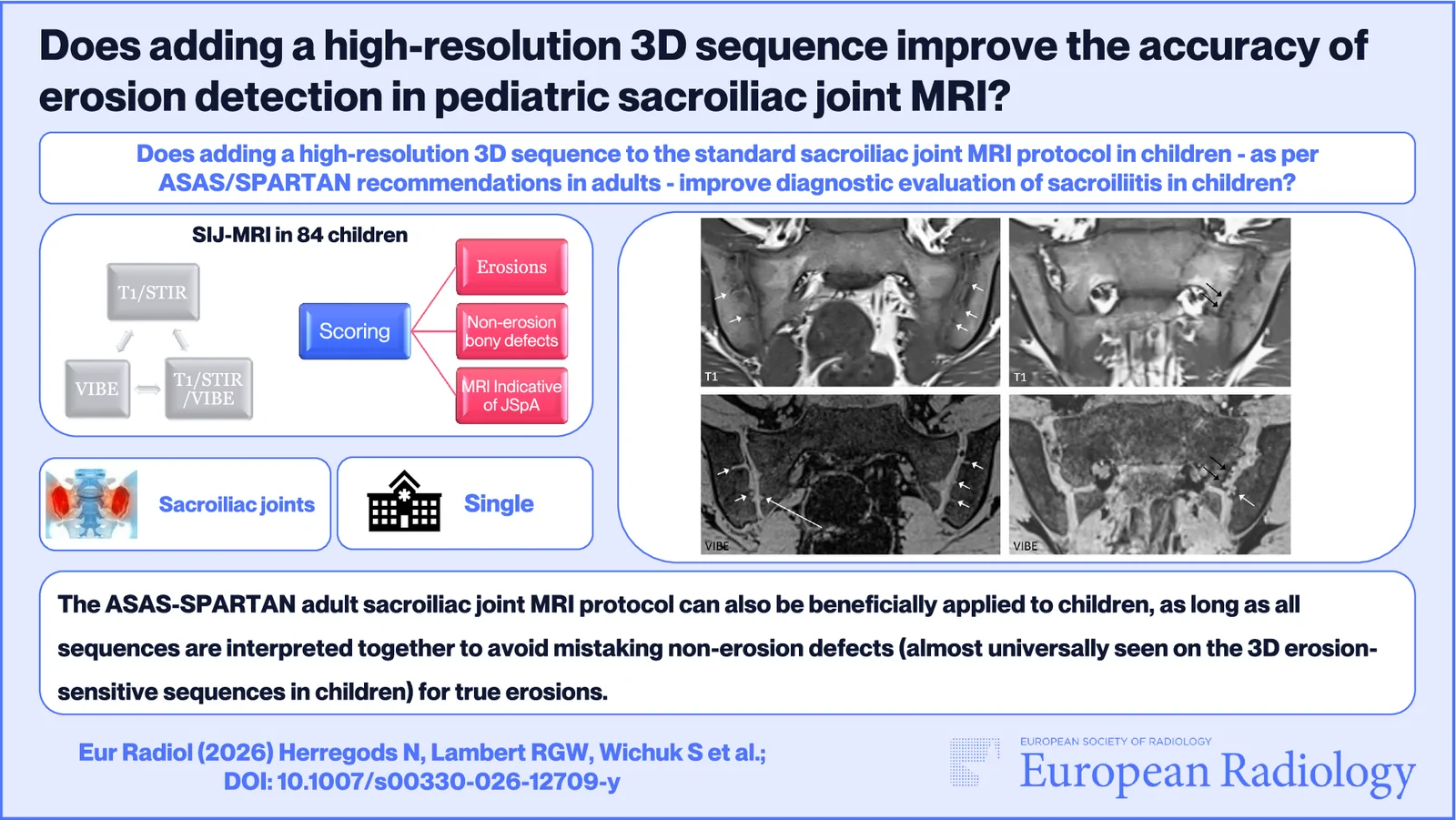

## Introduction

Magnetic resonance imaging (MRI) of the sacroiliac joints (SIJ) is the first-choice imaging modality for assessment of sacroiliitis, in adults and in children with possible Juvenile Spondyloarthritis (JSpA) [1–3]. The importance of identifying structural lesions such as erosions to enhance confidence in interpretation has been emphasized recently in adults [4–7].

It has been shown in adults that adding a specific sequence designed to optimally depict the joint line articular surface can enhance the detection of erosions and improve MRI utility [8–12]. For this reason, the standard protocol for SIJ MRI in adults recommended by the ASAS-SPARTAN collaboration now includes an erosion-specific sequence that is sensitive for the detection of erosion [13]. Due to a lack of adequate data, no similar recommendation regarding a pediatric SIJ MRI protocol has yet been made [13]. It is not yet clear whether adding an erosion-sensitive sequence in children has added value similar to adults.

In children, it remains notoriously difficult to assess inflammatory and structural lesions due to the presence of non-ossified cartilage and the absence of a fully developed subchondral bone plate in pediatric SIJ [14, 15]. Because of the high prevalence of confounding normal variations, T1-weighted spin-echo sequences are more challenging for the detection of erosion in pediatric SIJ than in adults [14]. Accurately distinguishing true erosion from normal variation is difficult, which may result in under- or overdiagnosis, and even harmful treatment with biologicals when MRI readers are not familiar with these normal variations in the appearances of the subchondral bone. Therefore, an erosion-sensitive sequence might also be useful in children [8–12].

We performed this study to gather evidence regarding the value of adding such an erosion-sensitive specific sequence to pediatric SIJ MRI. We added a volumetric interpolated breath-hold examination (VIBE) sequence to our institutional scan protocol in a cohort of children. We performed a multi-observer reading exercise to evaluate MRI detection of erosions indicative of JSpA, and MRI global detection of JSpA, without and with the VIBE sequence added.

## Materials and methods

### Study group

This prospective study was approved by the Ghent University Hospital Ethics Committee. Informed consent was obtained from all children and parents. Inclusion criteria included age < 18 years and clinical suspicion of JSpA. SIJ MRIs were obtained from January 2021 to December 2024. Exclusion criteria were insufficient image quality (e.g., motion artefacts), unexpected relevant findings on the MR that could influence the results (e.g., neoplasm), or declining to consent.

### Magnetic resonance imaging

MRI was performed using a body flexed array coil in a 3-T magnet (Prisma/Vida, Siemens Healthineers). Concordant with ASAS/SPARTAN recommendations, sequences included the following: semicoronal (along sacral long axis parallel to S2 vertebral body) T1-weighted turbo-spin-echo (T1) (slice thickness (ST) 3 mm, repetition time/echo time (TR/TE) 919/10 ms); semicoronal short-tau inversion recovery (STIR) (ST 3 mm, TR/TE/inversion time (TI) 4560/56/220 ms); and semiaxial STIR. For this study, a volumetric interpolated breath-hold T1-weighted 3D VIBE gradient-echo (GRE) sequence was added (ST 1.1 mm; TR/TE: 12.1/4.75).

### Image review

Three fellowship-trained radiologists (N.H.—pediatric, J.J.—pediatric musculoskeletal, R.L.—musculoskeletal), with 16, 17, and 32 years of experience, respectively, separately reviewed SIJ MRI of 84 cases using a custom web-based interface developed on the CARE-arthritis platform (www.carearthritis.com). Readers were blinded to demographic, clinical, and other imaging findings, and to each other’s readings. The following features were scored: the presence of erosions indicative of JSpA, the presence of non-erosion bony defects, defined as defects in the subchondral bone consistent with normal anatomical variation (and not indicative of JSpA), and finally, whether the global MRI findings were indicative of JSpA or not. Scoring for each of these items included a confidence interval ranging from −5 (definitely no) to +5 (definitely yes). Features were scored in 3 different exercises: (1) MRI including only semicoronal T1-weighted spin-echo and STIR sequences; (2) VIBE sequence alone; (3) all 3 sequences together (T1, STIR, VIBE). Cases were randomly re-sorted for each exercise. In the 3rd exercise, the reader was asked to score whether erosions were much or slightly better seen on T1 or VIBE, and to indicate possible reasons for any difference. After completion of the 3rd exercise, we resolved discrepancies in global JspA diagnosis by reviewing each discrepant case together in an in-person consensus session and decided on a final decision “global MRI findings were indicative of yes/no.” A discrepant case was defined as a case with no 3/3 agreement of all three readers for JSpA diagnosis on all sequences. Basic descriptive statistics were applied on the results both before and after the read-out. The reference standard was pediatric rheumatologist diagnosis of JSpA.

### Statistical methods

Basic descriptive statistics for the diagnostic value of the sequences (sensitivity, specificity, ICC, and K values) were calculated. Statistical analysis was performed in Medcalc (v.26.2.6, Medcalc software Ltd., ©1993–2025), SPSS (IBM SPSS Statistics for Windows, Version 26.0, IBM Corp.), and R (v. 3.6.1, 05-07-2019). Interobserver reliability was assessed using Cohen’s Kappa for reader pairs and using Light’s kappa for all raters [16, 17].

### Patient and public involvement

Patients and/or the public were not involved in the design, conduct, reporting, or dissemination plans of this research.

## Results

### Study group

SIJ MRI of 84 children (50 girls), mean age 13.3 years (range 6.4–17.9), were obtained from January 2021 to December 2024. Of these children, 15/84 (17.9%) were HLA-B27 positive, and 26/84 (31.0%) were eventually diagnosed with JSpA by their pediatric rheumatologists. Demographics are summarized in Table 1.

**Table 1:** Demographics of the study population

	Total patients (*N* = 84)	JSpA positive (*N* = 26; 31%)		JSpA negative (*N* = 58; 69%)	
		Girls	Boys	Girls	Boys
		(*N* = 11; 42%)	(*N* = 15; 58%)	(*N* = 39; 67%)	(*N* = 19; 33%)
Mean age	13.3 y	13.4 y	14.7 y	13.6 y	11.8 y
Age range	6.4–17.9 y	9.6–16.9 y	9.6–17.4 y	8.2–17.9 y	6.4–16.8 y
Clinical diagnosis of JSpA	26 (31%)	11 (22%)	15 (44%)	/	/
HLA-B27	15 (17..9%)	6 (55%)	9 (60%)	4 (10%)	1 (5%)

*N* number of participants, *JSpA* juvenile spondylarthropathy, *y* years

### MRI

**Fig. 1:**
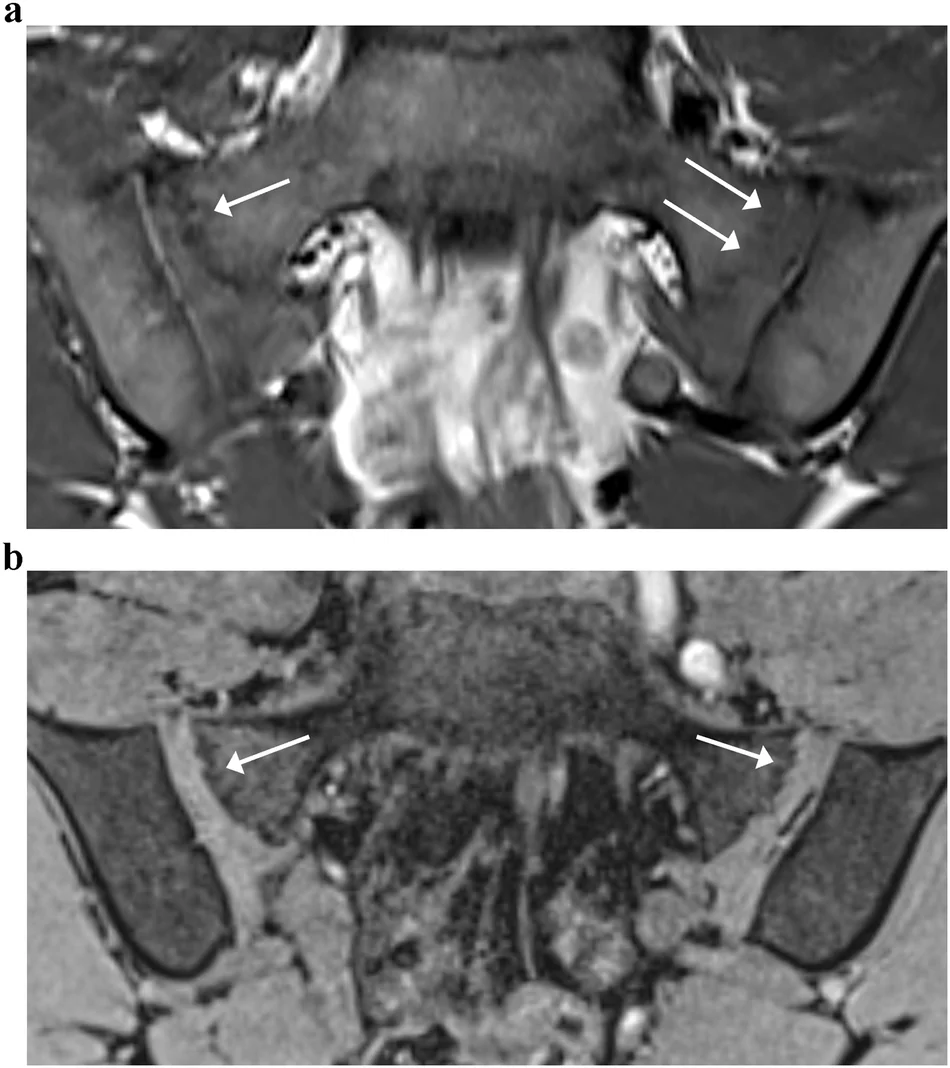
T1 (**a**) and corresponding VIBE (**b**) image illustrating the difficult joint delineation on T1 in pediatric SIJ, with blurred joint margins and irregularities (arrows), mainly on the sacral side. On the VIBE sequence, joint margins are clearly visible and sharp, and small irregularities on the sacral side of both SIJ (arrows) can be better depicted. T1, T1-weighted spin-echo; SIJ, sacroiliac joints; VIBE, volumetric interpolated breath-hold examination

**Fig. 2:**
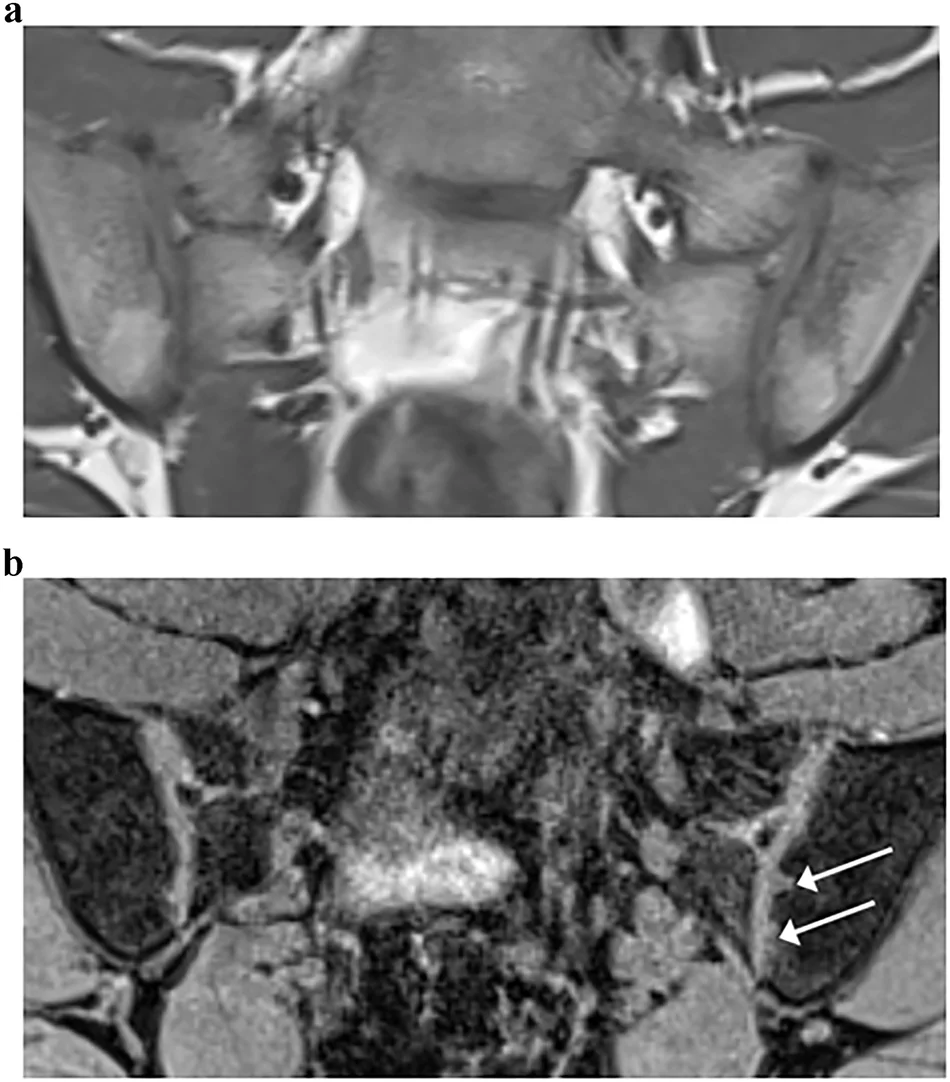
T1 (**a**) and corresponding VIBE (**b**) SIJ image illustrating better depiction of irregularities on VIBE than T1, which are mainly seen here on the iliac side of the left SIJ (arrows). No other features of sacroiliitis can be seen, so this is most likely normal variability. T1, T1-weighted spin-echo; SIJ, sacroiliac joints; VIBE, volumetric interpolated breath-hold examination

**Fig. 3:**
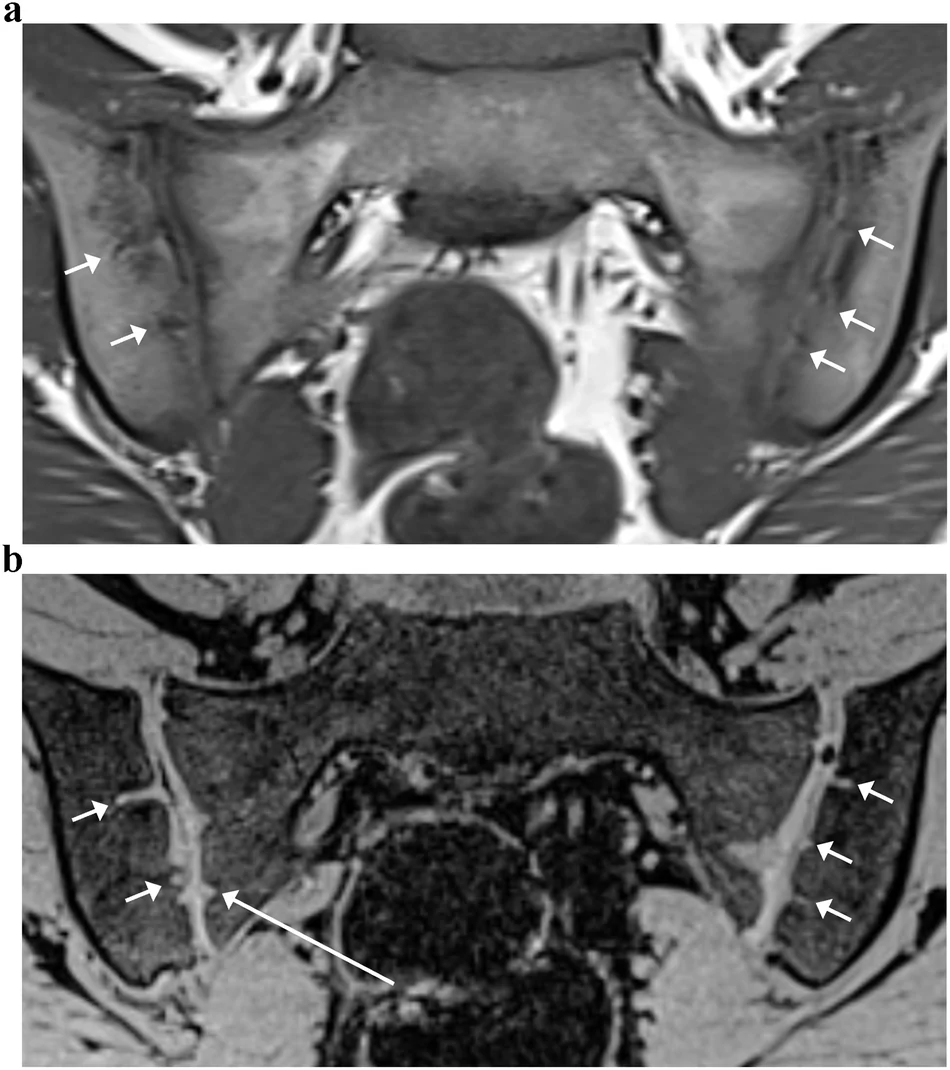
T1 (**a**) and corresponding VIBE (**b**) SIJ image illustrating the better visualization and increasing confidence in diagnosing non-erosion bony defects on VIBE vs T1 on the iliac side of both SIJ (short white arrows). There is also an additional bony defect visible on the sacral side of the right SIJ on VIBE, which is not visible on T1 (long white arrow). T1, T1-weighted spin-echo; SIJ, sacroiliac joints; VIBE, volumetric interpolated breath-hold examination

**Fig. 4:**
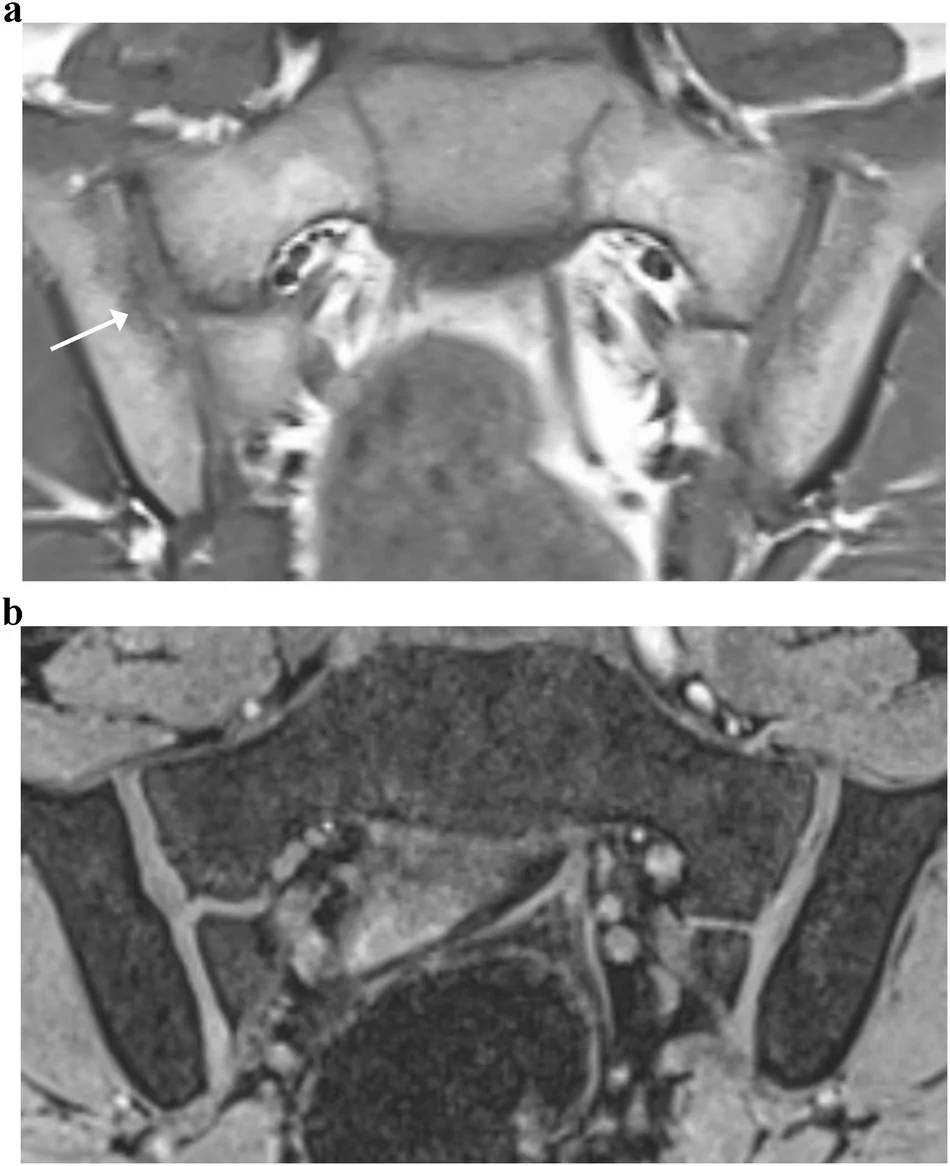
T1 (**a**) and corresponding VIBE (**b**) image of the SIJ illustrating the added value of the VIBE sequence for increasing confidence for diagnosing erosion. On T1, there is a possible small erosion on the iliac side of the right SIJ (arrow), but on the VIBE, the articular surface is smooth with minimal undulation, and this is clearly not an erosion. T1, T1-weighted spin-echo; SIJ, sacroiliac joints; VIBE, volumetric interpolated breath-hold examination

**Fig. 5:**
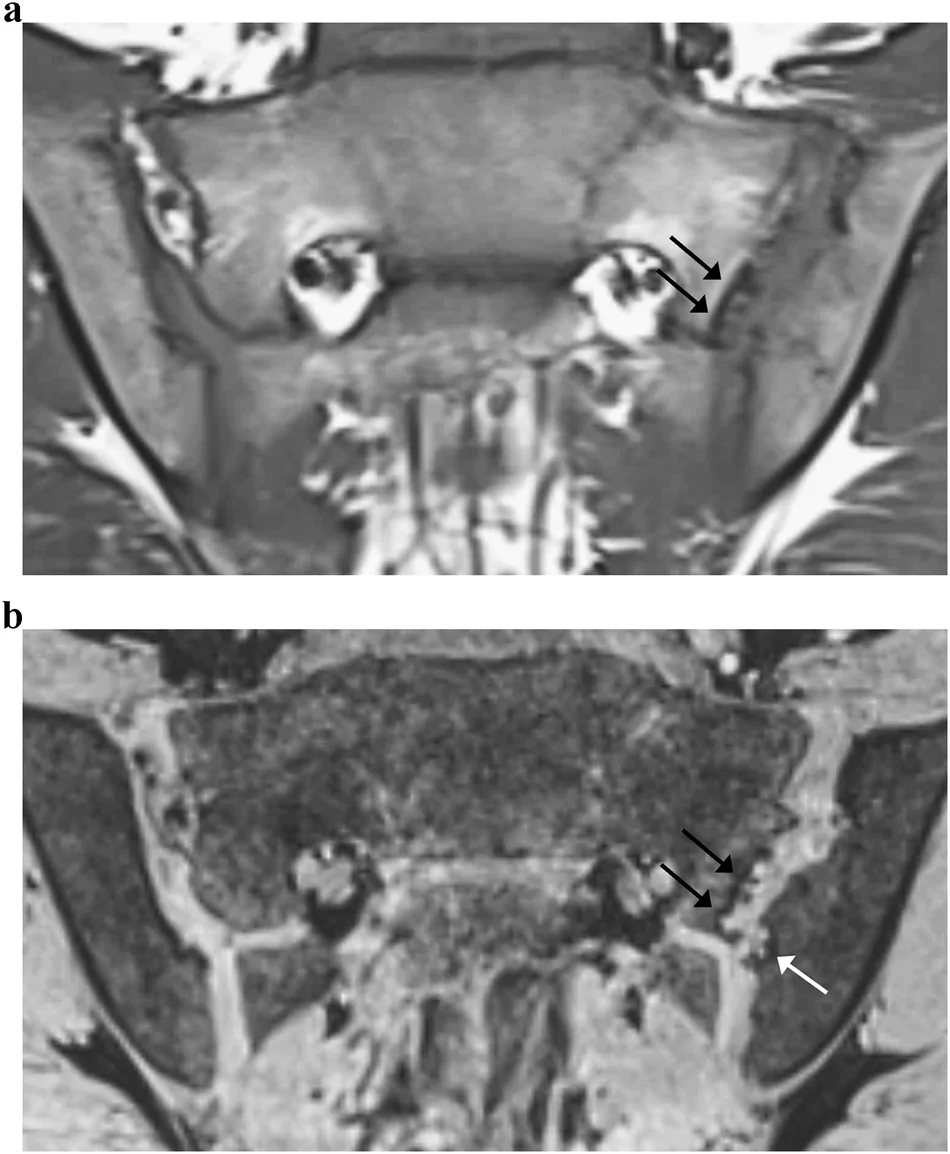
T1 (**a**) and corresponding VIBE (**b**) image of the SIJ illustrating the added value of VIBE in this case showing a row of definite erosions on the sacral side of the left SIJ (black arrows), with surrounding sclerosis. On the VIBE, however, there is also an erosion visible on the iliac side of the left SIJ (white arrow), which is not as clearly visible on T1. T1, T1-weighted spin-echo; SIJ, sacroiliac joints; VIBE, volumetric interpolated breath-hold examination

**Fig. 6:**
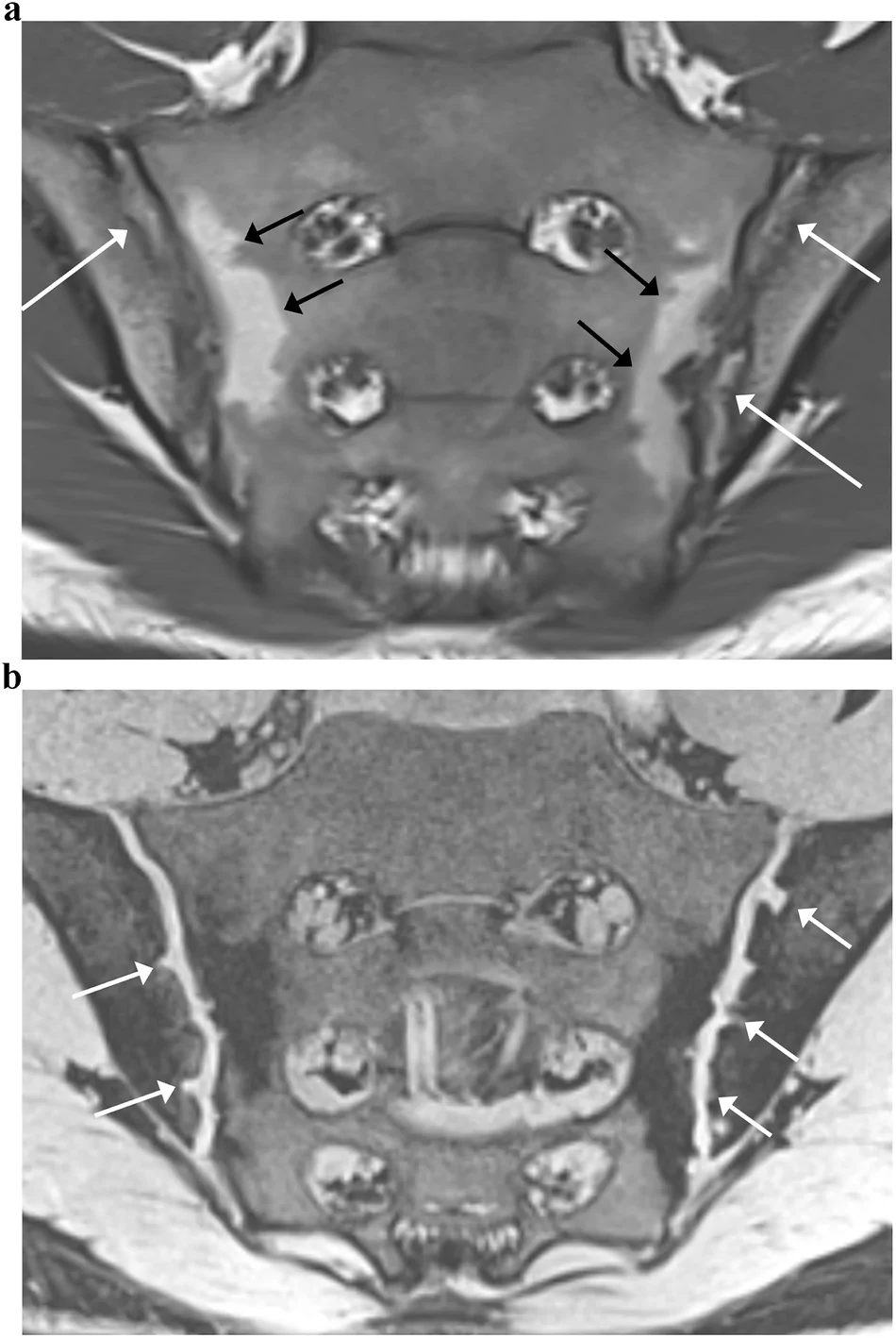
T1 (**a**) and corresponding VIBE (**b**) image of the SIJ illustrating the complementarity of T1 and VIBE. On T1, the fat lesion (black arrows) and backfill (long white arrows) can be better seen, whereas on the VIBE, the erosions (short white arrows) can be better depicted. T1, T1-weighted spin-echo; SIJ, sacroiliac joints; VIBE, volumetric interpolated breath-hold examination

‘Global impression MRI indicative of JSpA’ was scored as ‘yes’ in 14 cases (16.7%) on T1/STIR, in 21 cases (25.0%) on VIBE only, and in 15 cases (17.9%) on all sequences. Erosions were seen in 12 (14.3%), 22 (26.2%), and 13 cases (15.5%) for the 3 exercises; non-erosion bony defects were seen in 43 (51.2%), 79 (94.0%), and 80 (95.2%) of cases, respectively. In the 13 cases where erosions were present, erosions were better visualized on VIBE compared to T1 on 36/39 occasions (92%) (Figs. 1–5). The 3 readers only scored T1 as better for erosion detection than VIBE 3 times, twice because of fat surrounding the erosion, and once because of surrounding sclerosis (Fig. 6). The main reasons readers noted for preferring VIBE over T1 for erosion detection were higher in-plane resolution (25.6%), thinner slices (48.7%) or better joint line contrast (56.4%) on VIBE. Multiple reasons were given in most cases.

Analysis of the effect of age on erosion was hampered by the overall low prevalence of erosion, with limited effect on the observation of either erosion or non-erosion defect (Table 2). On T1 only, the proportion of subjects in which all three readers agreed that erosion was absent increased from 70.6% at age < 11 to 86.4% at age > 15. However, this trend was not observed on VIBE or on the ‘All Sequence’ reading exercise, where age had no significant effect on erosion. The only age effect on the observation of non-erosion bony defect was increasing reader agreement, which reached 100% in the older age group on the ‘All Sequence’ reading exercise.

**Table 2:** Inter-reader agreement for erosion and non-erosion bony defects on all three exercises (T1, VIBE, and all sequences) for different age groups

		Erosion			Non-erosion bony defects		
		T1/STIR	VIBE	All sequences	T1/STIR	VIBE	All sequences
Age group	3 readers	*n* (% within each age group)			*n* (% within each age group)		
< 11 years (*n* = 17)	Agree neg 3/3	12 (70.6%)	11 (64.7%)	12 (70.6%)	2 (11.8%)	0 (0%)	0 (0%)
	Disagree 1/2 or 2/1	4 (23.5%)	4 (23.5%)	4 (23.5%)	11 (64.7%)	3 (17.6%)	2 (11.8%)
	Agree pos 3/3	1 (5.9%)	2 (11.8%)	1 (5.9%)	4 (23.5%)	14 (82.4%)	15 (88.2%)
11–15 years (*n* = 45)	Agree neg 3/3	34 (75.6%)	27 (60.0%)	31 (68.9%)	5 (11.1%)	0 (0%)	0 (0%)
	Disagree 1/2 or 2/1	5 (11.1%)	14 (31.1%)	10 (22.2%)	31 (68.9%)	4 (8.9%)	5 (11.1%)
	Agree pos 3/3	6 (13.3%)	4 (8.9%)	4 (8.9%)	9 (20.0%)	41 (91.1%)	40 (88.9%)
> 15 years (*n* = 22)	Agree neg 3/3	19 (86.4%)	14 (63.6%)	16 (72.7%)	6 (27.3%)	2 (9.1%)	2 (9.1%)
	Disagree 1/2 or 2/1	1 (4.5%)	6 (27.3%)	4 (18.2%)	11 (50.0%)	2 (9.1%)	0 (0%)
	Agree pos 3/3	2 (9.1%)	2 (9.1%)	2 (9.1%)	5 (22.7%)	18 (81.8%)	20 (90.9%)
All ages (*n* = 84)	Agree neg 3/3	65 (77.4%)	52 (61.9%)	59 (70.2%)	13 (15.5%)	2 (2.4%)	2 (2.4%)
	Disagree 1/2 or 2/1	10 (11.9%)	24 (28.6%)	18 (21.4%)	53 (63.1%)	9 (10.7%)	7 (8.3%)
	Agree pos 3/3	9 (10.7%)	8 (9.5%)	7 (8.3%)	18 (21.4%)	73 (86.9%)	75 (89.3%)

*T1* T1-weighted spin-echo, *STIR* short-tau inversion recovery sequence, *VIBE* volumetric interpolated breath-hold examination, *n* number, *neg* negative, *pos* positive

All 3 readers separately agreed on the global impression whether MRI was indicative of JSpA in 58/84 cases (67%). For the other 28 cases (33%), disagreements were resolved at an in-person consensus read-out. In 24 cases, the majority of reader opinion was confirmed. However, after discussion, 3 cases changed from majority opinion positive to consensus negative, and 1 case changed from majority opinion negative to consensus positive.

### Clinical relevance

In general, SIJ MRI in children is highly specific for JSpA, but sensitivity is low compared to a rheumatologist’s diagnosis. On all sequences, before consensus read-out, the majority opinion scored 53 MRI scans negative (17 Male (M)/36 Female (F)) for JSpA and 5 cases positive (2 M/3 F) in the clinical JSpA negative group (91.4% specificity). In the clinical JSpA positive group, 16 MRI scans were scored negative (10 M/6 F), and only 10 cases were scored positive (5 M/5 F) (38.5% sensitivity). Sensitivity, specificity, and positive and negative predictive values (PPV and NPV) for all 3 exercises are summarized in Table 3. Mean absolute confidence scores were significantly better for all categories of observation on the ‘All sequences’ reading exercise compared to the ‘T1/STIR only’ exercise, and all improvements were statistically significant. Mean absolute values [0–5] of all confidence scores for the different features in the three different exercises are summarized in Table 4.

**Table 3:** Sensitivity, specificity, positive and negative predictive values (PPV and NPV), and accuracy for the diagnosis of JSpA on MRI for all 3 exercises

	T1/STIR	VIBE	All sequences	
			Before consensus read-out	After consensus read-out
Sensitivity (95% CI)	34.62% (17.2–55.7)	42.31% (23.4–63.1)	38.46% (20.2–59.4)	38.46% (20.2–59.4)
Specificity (95% CI)	91.38% (81.0–97.1)	82.76% (70.6–91.4)	91.38% (81.0–97.1)	94.8% (85.6–98.9)
PPV (95% CI)	64.3% (40.1–82.9)	52.4% (34.9–69.3)	66.7% (43.2–84.0)	76.9% (50.0–91.7)
NPV (95% CI)	75.7% (70.0–80.7)	76.2% (69.3–81.9)	76.8% (70.8–81.9)	77.5% (71.6–82.4)
Accuracy (95% CI)	73.8% (64.4–83.2)	70.2% (60.4–80.0)	75.0% (65.7–84.3)	77.4% (68.4–86.3)

*JSpA* juvenile spondyloarthropathy, *T1* T1-weighted spin-echo, *STIR* short-tau inversion recovery sequence, *VIBE* volumetric interpolated breath-hold examination, *CI* confidence interval, *PPV* positive predictive value, *NPV* negative predictive value

**Table 4:** Mean absolute confidence ratings including standard deviation (SD) for Global JSpA yes/no, for presence of erosion, and for presence of non-erosion bony defect for all 3 exercises, with indication of the *p*-value for statistical significance between T1/STIR vs all sequences

	T1/STIR	VIBE	All sequences	*p*-value
	Mean (SD)	Mean (SD)	Mean (SD)	T1/STIR vs All
Global JSpA yes/no	3.6 (0.81)	2.71 (0.84)	3.9 (0.71)	< 0.001
Erosion	3.67 (0.73)	3.11 (0.89)	3.87 (0.73)	0.015
Non-erosion bony defect	3.05 (0.52)	3.94 (0.53)	4.19 (0.45)	< 0.001

*JSpA* juvenile spondyloarthropathy, *T1* T1-weighted spin-echo, *STIR* short-tau inversion recovery sequence, *VIBE* volumetric interpolated breath-hold examination

### Inter-reader agreement

Inter-reader reliability for all features in all exercises is moderate to substantial, and increases significantly for non-erosion bony defects on T1/STIR vs All Sequences (0.18 to 0.51) but decreases for both erosion and global JSpA diagnosis after adding the VIBE sequence (0.71 to 0.53 and 0.61 to 0.39, respectively).

Inter-reader reliability for all features in all exercises is summarized in Table 5.

**Table 5:** Inter-reader reliability using Light’s kappa (with 95% confidence interval [CI]) for Global SpA yes/no, for presence of erosion, and for presence of non-erosion bony defect for all 3 exercises

	T1/STIR	VIBE	All sequences
	Kappa (95% CI)	Kappa (95% CI)	Kappa (95% CI)
Global JSpA yes/no	0.61 (0.58–0.64)	0.48 (0.46–0.5)	0.39 (0.37–0.42)
Erosion	0.71 (0.69–0.73)	0.49 (0.48–0.5)	0.53 (0.49–0.56)
Non-erosion bony defect	0.18 (0.17–0.2)	0.47 (0.45–0.49)	0.51 (0.48–0.54)

*JSpA* juvenile spondyloarthropathy, *T1* T1-weighted spin-echo, *STIR* short-tau inversion recovery sequence, *VIBE* volumetric interpolated breath-hold examination

## Discussion

This study aimed to determine whether the standard adult MRI SIJ scan protocol recommended by the ASAS-SPARTAN collaboration [13] can be applied to children and assess the value of adding an erosion-sensitive VIBE GRE sequence in pediatric SIJ MRI. We specifically evaluated performance in the detection of erosions, distinguishing erosions from non-erosion defects, and diagnosing JSpA. We found that non-erosion bony defects are seen in nearly all children on the VIBE sequence, which was challenging to interpret in isolation. Adding VIBE to the standard pediatric SIJ MRI scan protocol significantly improved reader confidence in detecting erosions and diagnosing JSpA, improved reliability of identifying non-erosion bony defects, and also showed a trend toward increased sensitivity and specificity for diagnosing JSpA.

Assessing erosion in SIJ in children is notoriously difficult due to the normal ossification process, and T1-weighted spin-echo sequences are more challenging for the interpretation of erosion in pediatric SIJ than in adults [14, 15] (Figs. 1, 2). However, even in adults, interobserver agreement for erosion is at best moderate [18–20]. A specific sequence designed to optimally depict the articular surface can increase sensitivity and reader confidence for the detection of erosion, sclerosis, and ankylosis in adults [8–12], but no such studies have been published in children. In our study, experienced readers considered VIBE to be better able to detect erosions than T1 spin-echo in 92% of cases, because of thinner slices (3 mm vs 1 mm), higher resolution, and/or better articular contrast on the VIBE sequence. This is concordant with previous adult SIJ studies, which also noted the narrower slice thickness as the main reason for superior erosion detection via high-resolution GRE sequences [8–12]. In three cases, T1 was considered better for erosion detection because fat or sclerosis surrounding the bony defect confirmed that the observation was more likely to be erosion rather than a non-erosion bony defect.

The VIBE sequence demonstrated more bony defects, both erosion and non-erosion, than the T1 sequence, with nearly all children (94%) having at least one non-erosion bony defect in their SIJ—nearly twice as many as on T1 (51%). When read in isolation, the VIBE sequence was associated with higher sensitivity, but lower specificity (42.3%/82.7%) compared to T1 alone (34.6%/91.4%) or to all sequences together (38.4%/91.4%). The VIBE sequence is difficult to interpret in isolation because the T1 sequence is essential for identification of other features of structural sacroiliitis, especially fat and sclerosis, while the water-sensitive STIR sequence helps distinguish erosion from normal anatomical variation by highlighting active inflammation surrounding the bony defect. Consequently, it is imperative that each sequence be interpreted in the context of information available on all other sequences, and not in isolation. Rigorous application of this principle should help less experienced readers avoid misinterpreting the plethora of non-erosion bony defects seen on VIBE as subchondral erosion and mitigate the likelihood of overdiagnosis of SpA.

Adding a VIBE sequence significantly improved inter-rater reliability for identification of non-erosion bony defects (Kappa coefficient up from 0.18 to 0.51) and also increased reader confidence, highlighting an important benefit of the high-resolution sequence (Figs. 4–6). While this increase in reader confidence did not yield a statistically significant improvement in diagnostic accuracy, the higher diagnostic certainty may hold clinical value in terms of workflow efficiency as it can potentially streamline clinical decision-making; more confident MRI negative examinations may reduce the need for redundant confirmatory sequences, for example adding contrast-enhanced sequences, repeat-scans, or ‘defensive’ specialist referrals; more confident MRI positive results may facilitate treatment decisions by rheumatologists in cases of clinical uncertainty.

However, despite improved reader confidence, inter-reader reliability for erosion and global JSpA diagnosis decreased when VIBE was added. This might be because even experienced readers are less familiar with appearances of pediatric SIJ on the relatively new VIBE sequence compared to T1 and STIR sequences, which have been used for decades. A similar point was noted by Krabbe et al [8], who suggested that performance statistics may further improve as readers become more acquainted with these methods.

A major confounding factor is the lack of validated definitions, especially for high-resolution 3D sequences, in both adults and children, and a lack of pediatric-specific definitions for lesions on T1/STIR. While there has been international consensus on how to define sacroiliac joint erosion on MRI in children [21, 22], this definition relies heavily on the original ASAS definition for this feature in adults, is only applicable on T1-weighted spin-echo, and does not apply to any other sequence. The appropriateness of applying this definition to children has been questioned [23]. There is also no consensus on what constitutes a ‘non-erosion bone defect’ contributing to low agreement for non-erosion bony defects on T1. In our study design, we chose not to create pediatric definitions in advance because we would have had to decide if any new definition applied to T1 only, VIBE only, or both, and there is no ‘global’ definition for SIJ erosion in adults that might have informed these decisions. A pediatric-specific definition for erosion would be helpful because some non-erosion bony defects/irregularities that are well visualized with VIBE would be considered erosions according to strictly applied adult definitions [18, 23]. The absence of available reference figures, training, or calibration is reflected in the moderate inter-reader reliability for scoring SIJ structural lesions and is concordant with results in many previous studies in both children and adults [8, 18, 19, 21].

Our study has several limitations. There is a broad lack of reference standards and validated pediatric-specific MRI definitions in this area, as discussed above. This was a single-center study, in which the overall population and number of positive cases were limited, reflecting daily clinical practice in a tertiary center. There may have been a learning or carry-over effect across reading sessions. However, this is likely to have been small because readers were blinded to clinical information and each other’s scoring, and cases were re-randomized between sessions. In fact, the carry-over effect could have been deleterious since the VIBE sequence was initially scored in isolation, and without any feedback or training, observers may have been mystified by seeing a feature on the high-resolution sequence that they had never seen before but were still forced to decide if it was an erosion or not. Prevalence of erosion was relatively low, which limited the utility of subgroup analysis including stratifying by patient age.

Clinician expert diagnostic opinion was the reference standard, as is common in studies in this field. This is inherently subjective and susceptible to bias because diagnostic assessments vary according to individual clinician experience, local diagnostic conventions, and awareness of clinical, laboratory, or imaging findings, particularly in the absence of an objective gold standard. Overreliance on MRI results may lead to an overestimation of MRI diagnostic accuracy, whereas overreliance on other clinical parameters may result in underestimation of MRI performance. These sources of bias are often amplified in single-center studies, where diagnostic decisions are shaped by a limited number of experts and a relatively homogeneous patient population. In this situation, the observed sensitivity and specificity must be interpreted as measures of clinical correlation rather than absolute diagnostic truth. Since the reference standard itself is subject to interobserver variability and potential misclassification, the reported diagnostic yields may either over- or understate the test’s true utility and raise doubt about the study’s internal validity. These considerations highlight the need for future multi-center studies, which would allow for greater heterogeneity in clinical practice and patient characteristics. Validation against longitudinal outcomes is warranted, including independent evaluation of data by central radiology and rheumatology evaluators of both clinical and imaging information, which would provide a more robust assessment of diagnostic performance and enhance the external validity of future results.

In conclusion, adding a VIBE sequence to the standard pediatric SIJ MRI protocol significantly increased reader confidence in MRI diagnosis of JSpA, with a non-significant trend toward improved sensitivity and specificity for diagnosing JSpA. The new sequence reliably demonstrated near-universal prevalence of non-erosion bony defects in children. Accordingly, we recommend that the ASAS-SPARTAN adult SIJ MRI protocol can be beneficially applied to children, with the following caveat: high-resolution erosion-sensitive sequences must always be interpreted together with T1 and STIR sequences to ensure that non-erosion defects in subchondral bone, which are very frequent in children and may be particularly conspicuous on high-resolution sequences, are not mistaken for erosions.

## Abbreviations

ASAS-SPARTAN: Assessment of SpondyloArthritis International Society (ASAS) and SpondyloArthritis Research and Treatment Network (SPARTAN)
CI: Confidence interval
GRE: Gradient-echo sequence
JSpA: Juvenile spondyloarthritis
MRI: Magnetic resonance imaging
N: Number of participants
NPV: Negative predictive value
PPV: Positive predictive value
SD: Standard deviation
SIJ: Sacroiliac joint
ST: Slice thickness
STIR: Short-tau inversion recovery sequence
T1: T1-weighted spin-echo sequence
TR/TE: Repetition time/echo time
VIBE: Volumetric interpolated breath-hold examination
y: Years
